# Nitrogen Forms Influence Microcystin Concentration and Composition via Changes in Cyanobacterial Community Structure

**DOI:** 10.1371/journal.pone.0085573

**Published:** 2014-01-10

**Authors:** Marie-Eve Monchamp, Frances R. Pick, Beatrix E. Beisner, Roxane Maranger

**Affiliations:** 1 Groupe de Recherche Interuniversitaire en Limnologie et en Environnement Aquatique (GRIL), Département de Sciences Biologiques, Université de Montréal, Montréal, Québec, Canada; 2 Center for Advanced Research in Environmental Genomics, Department of Biology, University of Ottawa, Ottawa, Ontario, Canada; 3 Groupe de Recherche Interuniversitaire en Limnologie et en Environnement Aquatique (GRIL), Department of Biological Sciences, University of Quebec at Montreal, Montréal, Québec, Canada; University of New South Wales, Australia

## Abstract

The eutrophication of freshwaters is a global health concern as lakes with excess nutrients are often subject to toxic cyanobacterial blooms. Although phosphorus is considered the main element regulating cyanobacterial biomass, nitrogen (N) concentration and more specifically the availability of different N forms may influence the overall toxicity of blooms. In this study of three eutrophic lakes prone to cyanobacterial blooms, we examined the effects of nitrogen species and concentrations and other environmental factors in influencing cyanobacterial community structure, microcystin (MC) concentrations and MC congener composition. The identification of specific MC congeners was of particular interest as they vary widely in toxicity. Different nitrogen forms appeared to influence cyanobacterial community structure leading to corresponding effects on MC concentrations and composition. Total MC concentrations across the lakes were largely explained by a combination of abiotic factors: dissolved organic nitrogen, water temperature and ammonium, but *Microcystis* spp. biomass was overall the best predictor of MC concentrations. Environmental factors did not appear to affect MC congener composition directly but there were significant associations between specific MC congeners and particular species. Based on redundancy analyses (RDA), the relative biomass of *Microcystis aeruginosa* was associated with MC-RR, *M. wesenbergii* with MC-LA and *Aphanizomenon flos-aquae* with MC-YR. The latter two species are not generally considered capable of MC production. Total nitrogen, water temperature, ammonium and dissolved organic nitrogen influenced the cyanobacterial community structure, which in turn resulted in differences in the dominant MC congener and the overall toxicity.

## Introduction

Lakes and coastal ecosystems around the world are subject to anthropogenic eutrophication, where excess loading of phosphorus (P) and nitrogen (N) results in ecosystem degradation with negative consequences on human health and regional economies [1]–[3]. Lake eutrophication is associated with an increase in algal biomass, as well as a shift in community structure with cyanobacteria often dominating and causing unsightly and odorous surface scums [1], [4]. Dense blooms furthermore increase turbidity and upon decomposition cause oxygen depletion in the water column affecting the entire food web [5]. Cyanobacteria also perform two important functional roles in ecosystems: some species are capable of fixing atmospheric N [6], [7] and some produce toxins harmful to humans and wildlife [8], [9]. Eutrophic lakes dominated by cyanobacteria often have low N to P ratios [10], [11] and the ability of cyanobacteria to offset N deficits has been argued as a reason to focus primarily on reducing P inputs to counter eutrophication in freshwater systems [12]. However, ratios are often poorer predictors of total cyanobacterial biomass than total phosphorus (TP) or total nitrogen (TN) concentrations [13], [14] and rates of N-fixation do not necessarily offset N limitation [15], [16].

In terms of toxin production, the most ubiquitous cyanotoxin in freshwater systems is the family of hepatotoxic compounds known as microcystins (MCs) which are cyclic heptapeptides that can be lethal to mammals if ingested [8], [17]. The microcystins are comprised of over 89 congeners, differing mainly in their amino acid composition as well as in the conformation and methylation of the molecule [18], [19]. MCs are typically reported as total concentrations [18], and few studies have examined the distribution of different MC congeners among lakes. Congener type is an essential consideration because the dominance of one variant over another in a bloom event will influence overall toxicity [20]. For example, LD_50_ toxicological studies on mice have shown that MC-LR and -LA are equally toxic, but are up to 12 times more toxic than other common variants such as -RR [18]. MC-YR is almost as toxic as -LR and -LA, and the demethylated form of MC-LR (MC-7dmLR) is five times less toxic than the methylated variant. It is therefore important to not only understand what factors may regulate overall MC concentrations but to understand what variables influence congener type.

Several cyanobacterial taxa can synthesize MCs, including *Microcystis* (Chroococcales), *Anabaena* (Nostocales), and *Planktothrix* (Oscillatoriales) [21] and of these genera, only *Anabaena* can fix atmospheric N. Hence, increases in the biomass of MC producing species *Microcystis*, *Anabaena* and *Planktothrix* spp. are typically associated with elevated total MC concentrations in cross-system comparisons [22]–[24]. However, when environmental predictors are considered in comparative studies, interestingly, total nitrogen (TN) is one of the strongest predictors of total MC concentrations and this response appeared essentially linear [22], [25] or showed a peaked relationship at intermediate levels of TN [26]. Furthermore, MC concentrations are highest in lakes with intermediate TN to TP ratios [2], suggesting that N availability in P rich systems could play a mitigating role in toxin production.

Indeed, several experimental and a few *in situ* studies have shown that cyanobacteria appear to out-compete other algal species for reduced N forms such as ammonium (NH_4_^+^) and urea [27]–[30], and independent experimental work has also linked N availability to toxin production [31], [32]. A recent mesocosm study examining the effects of urea, and nitrate (NO_3_^−^) in a hypereutrophic lake found that under high P, the addition of both NO_3_^−^ and urea increased total MC concentration by 10 to 13-fold [33], [34]. However, this study did not assess the influence on congener type. A few culture experiments suggest that congener composition may be influenced by N availability as well given that some congener types are more N-rich such as MC-RR which is composed of two molecules of arginine [23], [35], but evidence for this influence in natural systems is limited.

Although several lines of evidence point to N as important for cyanobacterial toxicity, the way N and its different forms affect the composition of cyanobacterial communities *in situ* and their ability to produce different MC congeners remains an open question. The aim of this study was to test, in natural ecosystems, the hypothesis that different N forms, alone or in combination with other environmental variables, influence the cyanobacterial community structure, the MC concentration, as well as the MC congener composition.

## Methods

### Study Sites

The study lakes were all located in the Eastern Townships in south-western Quebec, Canada. Lakes were selected based on their history of cyanobacterial blooms [22], [25] and their trophic status ranging from meso-eutrophic to hypereutrophic according to OECD (Organisation of Economic Cooperation and Development) criteria [36]. The selected lakes are relatively small, shallow, and differ in their catchment characteristics (Table 1). Lake Bromont is located in a hilly and largely forested watershed. It is a meso-eutrophic lake and the deepest of the study lakes (maximum depth 7.5 m); it is thus the only one that thermally stratifies during the summer months. Lake Waterloo is eutrophic and polymictic, with a predominantly forested watershed too, and with the highest population density (157 ind.km^−2^). Petit Lac St. François (PSF) is hypereutrophic, polymictic and has an agricultural watershed. It is the shallowest of the study lakes and has the shortest water residence time (Table 1).

**Table 1:** Morphological and physical characteristics for the three study lakes.

Lake	Bromont	Waterloo	Petit Lac St. François
Mean depth (m)	4.0	2.7	1.1
Maximum depth (m)	7.5	4.8	1.8
Water temperature range (°C)	14.8–23.1 (epi)	15.9–24.7	15.1–27.8
	10.6–14.6 (meta)		
Watershed area (km^2^)	24.8	28.7	19.5
Lake surface area (km^2^)	0.46	1.47	0.87
Lake volume (km^2^)	1.87·10^6^	4.03·10^6^	9.74·10^5^
Water residence time (days)	39	116	22
Population density (ind·km^−2^)	35	157	54
Forested area (%)	72.6	65.9	22.6
Agricultural area (%)	6.7	10.1	47.9

epi: epilimnion.

meta: metalimnion.

### Sample Collection

We sampled each lake on a monthly or fortnightly basis from May to October 2010 resulting in a time series with eight data points per lake and four additional points in the metalimnion of Bromont. Light penetration was measured using a Li-Cor photometer (Li-189) and light extinction coefficients (*k*) were calculated using the Lambert-Beer law [37]. Water temperature profiles were obtained using a multiprobe sonde (YSI 556-MPS) at 0.25 m intervals. Integrated water samples of the mixed (epilimnetic) layer were collected near midday at the centre of each lake using a Tygon tube and samples were transferred to 4 L acid-washed dark plastic bottles pre-conditioned with lake water. When Lake Bromont was stratified, we observed a metalimnetic chlorophyll a peak using a Fluoroprobe (BBE-Moldaenke). An additional sample was taken at that depth using a horizontal 2 L Van Dorn bottle. Whole water samples for phytoplankton identification and enumeration were preserved with Lugol’s iodine in 125 mL flint glass bottles in the field. Samples for MC analysis were filtered promptly in the field using a peristaltic pump (Masterflex L/S 7519-06, Cole-Parmer) at 80–100 mL·s^−1^ onto pre-ashed and pre-weighed 47 mm Whatman GF/C filters. After filtration of 200 to 1,000 mL of lake water (or until filter clogged), filters were individually folded into 2 mL cryogenic tubes (Simport, Beloeil, Canada) and immediately flash-frozen in liquid nitrogen. Thereafter, samples were stored at −20°C in the dark until MC analysis.

Unfiltered water samples for TN and TP analyses were stored in 100 mL acid-washed glass tubes. For total dissolved nitrogen (TDN) and soluble reactive phosphorus (SRP), 50 mL of water was syringe-filtered through 0.45 *μ*m Acrodisc® filters (Pall Corporation) and stored in the same manner as for TN and TP. Water for NH_4_^+^, NO_3_^−^ and nitrite (NO_2_^−^) analyses was also filtered through 0.45 *μ*m Acrodisc® filters and stored in 125 mL acid-washed Nalgene® bottles. All samples for nutrient analyses were either analyzed promptly upon return to the lab or stored frozen until processing.

### Microcystin Analyses

Prior to extraction, filters were oven-dried overnight at 60°C, and weighed. MC extraction was performed using a pressurized liquid extraction method with an accelerated solvent extractor (ASE 200, Dionex Corporation instrument, Bannockburn, IL, USA) at 60°C and 14 MPa [38]. In brief, dried filters were unfolded and inserted in cylindrical stainless steel cells (11 mL) packed with prewashed Hydromatrix®. Extracts recovered in 75% methanol (MeOH) were collected in amber vials previously rinsed with HPLC-grade solvent. Samples were then evaporated under a gentle ultra-high purity N flow using a Zymark Turbovap® II Concentration Workstation and re-suspended in 50% MeOH in ultra-pure water. Recovered samples were filtered through pre-conditioned 0.22 *μ*m Acrodisc® filters (Pall Corporation) for purification prior to HPLC analysis of individual congeners. Further dilutions (50% MeOH in ultra-pure water) were done if necessary for the ELISA test for total microcystins.

Total MC concentration was measured with an ELISA kit for microcystin (Quantiplate®, Envirologix, ME, USA). This is a direct competitive ELISA using a polyclonal antibody bound to a microtiter plate. MC-LR was used as a standard; therefore all measurements were expressed in MC-LR equivalents. Each test was run in duplicate and samples with coefficient of variation (CV) values over 15% were reanalyzed, according to the manufacturer’s recommendation.

Because ELISA does not distinguish between congeners, we analyzed the same extracts using a reverse-phase high-pressure liquid chromatography (RP-HPLC) with UV-detection equipped with a photodiode array (PDA) for the quantification of five MC congeners [38]. We used a HP series 1100 HPLC-PDA operated at 40°C, with a Zorbax Eclipse XDB-C18 column. The flow rate was of 0.5 mL per minute with a solvent gradient composition from 90% water and 10% acetonitrile, reaching 100% acetonitrile in 43 minutes. A volume of 0.05% trifluoroacetic acid, commonly used as a pairing agent in reverse-phase HPLC peptide separation, was added to both acetonitrile and water. UV spectra between 200 nm and 300 nm were collected and concentrations were calculated from the absorbency at 239 nm by comparison against purified extracts of certified standards of MC-RR, -YR, -LR, -7dmLR and -LA, and nodularin (all obtained from Cedarlane and the National Research Council, Halifax, Canada). Nodularin was used as an internal standard and spiked prior to extraction to verify recovery, which averaged 74.5%. In addition to the five MC congeners above, we also assessed the presence of four other common congeners (-WR, -LY, -LW, -LF) for which standards are available by examining their corresponding retention times on chromatographs. None of these congeners were detected in the study lakes.

### Phytoplankton Analyses

Total phytoplankton biomass was calculated from counts of cells greater than 2 *μ*m using a Zeiss AXIO A1 inverted microscope at X200, and X400 magnification. Aliquots (7 mL) of preserved phytoplankton sample were allowed to settle overnight in a 26 mm diameter chamber according to the Utermöhl method [39] before microscopic identification, cell measurements and enumeration. Phytoplankton biomass was estimated by converting cell volume to biomass assuming a specific density of 1 g·cm^−3^, which is, by convention, used for all phytoplankton taxa. The computer counting software Algamica (version 4.0) was used [40] for counting and estimations of cell biovolumes using assigned geometric shapes dimensions. Total volumetric biomass (*μ*g·L^−1^) for all cells >2 *μ*m as well as for cyanobacterial biomass by species (when possible) were calculated for each sample. In the case of *Microcystis*, we were able to identify two species (*M. aeruginosa* and *M. wesenbergii*), but in some samples, individual cells could not be identified to species and were referred to as *Microcystis* spp.

### Nutrient Analyses

Nutrient concentrations (NO_3_^−^, NO_2_^−^, NH_4_^+^, TP and SRP) were measured spectrophotometrically according to standard techniques [41]. NH_4_^+^ was measured using the phenol-hypochlorite method relying on estimations of an indophenol blue compound after reaction with phenol and hypochlorite [42]. Total dissolved nitrogen (TDN), NO_3_^−^ and NO_2_^−^ were all measured in the same manner, although TDN was first treated with a persulfate digestion to convert to NO_3_^−^, and all NO_3_^−^ was then reduced to NO_2_^−^ using a cadmium coil prior to analysis. NO_2_^−^ concentration was measured with the Griess reaction. Briefly, sulfanilamide and N-naphthyl-ethylenediamine were successively added to a sample to form a stable azo compound that can be compared with a calibration curve treated in the same manner [42].

Dissolved organic nitrogen (DON) concentration was obtained by subtracting NO_3_^−^, NO_2_^−^ and NH_4_^+^ from TDN with an estimated standard deviation (SD) for [DON] incorporating measurement error (*S* ^2^) propagation according to SD_DON_ = (*S* ^2^_TDN_+*S* ^2^_NH4_+*S* ^2^_NO3+NO2_)^1/2^ [43]. TN concentration was estimated as the sum of TDN and suspended particulate nitrogen (SPN) with SD_TDN_ calculated similarly to SD_DON_. The CV between sample replicates was less than 10% in all cases. TP and SRP measurements were done with the molybdenum blue method using an Astoria analyzer (Astoria-Pacific, Clackamas, OR).

### Statistical Analyses

Data were examined to determine whether they met normality assumptions using a Shapiro-Wilks test and were transformed (log_10_ or square root) when necessary. All statistical analyses were performed using *R* version 2.14 [44]. Multiple linear regressions were used to determine which environmental and taxonomic factors could best predict the observed differences in MC concentrations among lakes and dates. For multivariate comparisons of the MC congener composition with environmental variables and cyanobacterial community structure (based on taxon biomasses), we used principal components analysis (PCA) and redundancy analysis (RDA). Species biomass data were Hellinger-transformed as recommended for species linear ordinations [45], and as this transformation ensures unbiased estimation of variation partitioning of explanatory components in RDA [46]. Forward stepwise selection of explanatory variables, based on Akaike information criterion (AIC), was used to determine the best regression models. To test the significance of the RDAs, permutation tests on the models were performed with a minimum of 999 permutations, using the *anova.cca* function of the *Vegan* package in *R*.

## Results

### Nutrient Concentrations

**Figure 1:**
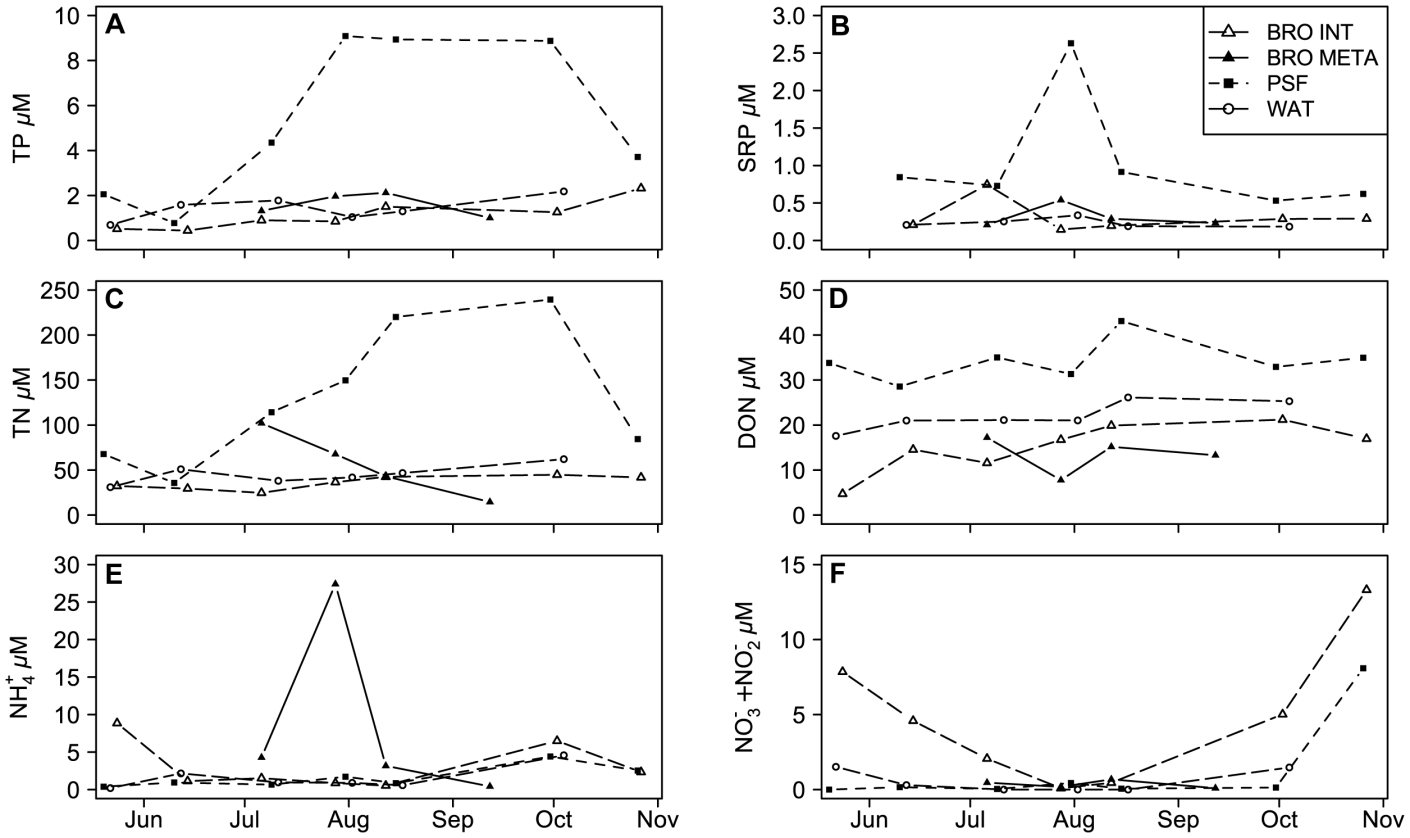
Concentration of nutrients (*μ*M) from April to October in the three study lakes. A) TP, B) SRP, C) TN, D) DON, E) NH_4_^+^, and F) NO_3_^−^+NO_2_^−^. BRO INT, integrated samples from the epilimnion of lake Bromont; BRO META, samples from the metalimnion of lake Bromont; PSF, Petit Lac St. François; WAT, Lake Waterloo.

TP was high in all lakes. In the meso-eutrophic Lake Bromont, TP varied seasonally from 0.55 to a high of 2.32 *μ*M observed in autumn (Fig. 1A) with a mean value of 1.1 *μ*M. Waterloo had an average concentration of 1.84 *μ*M, ranging from 1.04 to 5.17 *μ*M. Petit St. François (PSF) was much more eutrophic with a maximum TP of 11.13 *μ*M sustained from the beginning of August. Soluble reactive phosphorus (SRP) was measureable and relatively constant in all lakes with the exception of a peak in PSF, probably due to internal loading in this polymictic system (Fig. 1B).

TN was also high in all lakes, with average concentrations ranging from 24.69 *μ*M (Bromont epilimnion) to 239.46 *μ*M (PSF) (Fig. 1C). DON concentrations were highest in PSF with a mean of 33.37 *μ*M, followed by Waterloo (mean 21.57 *μ*M) and Bromont (means of 14.91 and 13.28 *μ*M for epi- and metalimnion respectively). The DON concentration was relatively stable over the summer sampling period in all lakes (Fig. 1D), accounting for an average of 32.5% of TN. A range of NH_4_^+^ concentrations were observed across all three lakes (1.59 to 9.51 *μ*M). Higher concentrations and an obvious peak occurred in the metalimnion of Lake Bromont at the depth just above the anoxic hypolimnion (Fig. 1E).Nitrite and nitrate (NO_2_^−^+NO_3_^−^) decreased towards the end of spring in Bromont and Waterloo and increased again at the end of summer. In the metalimnion of Bromont, NO_2_^−^+NO_3_^−^ remained low until the lake destratified. PSF was depleted in NO_2_^−^+NO_3_^−^, even in early spring, and the concentrations remained low to virtually undetectable throughout summer until they increased in October (Fig. 1F).

All lakes had relatively low mean TN: TP ratios, ranging between 8.7 and 15.2, except for Bromont’s metalimnion where the ratio was 28. Overall, mean DIN: SRP ratios were slightly higher ranging from 14 in PSF and Bromont (epilimnion) to 27 in Waterloo. pH varied among lakes, being on average highest in hypereutrophic PSF at 9.05 (range: 7.53–9.53) and in Waterloo at 8.01 (range: 7.08–8.96) and lowest in both the epilimnion (7.33) and metalimnion (7.15) of Bromont. Accurate pH measurements were only done on the last five sampling dates; therefore pH was not included in subsequent statistical analyses.

### Cyanobacterial Community Composition

**Figure 2:**
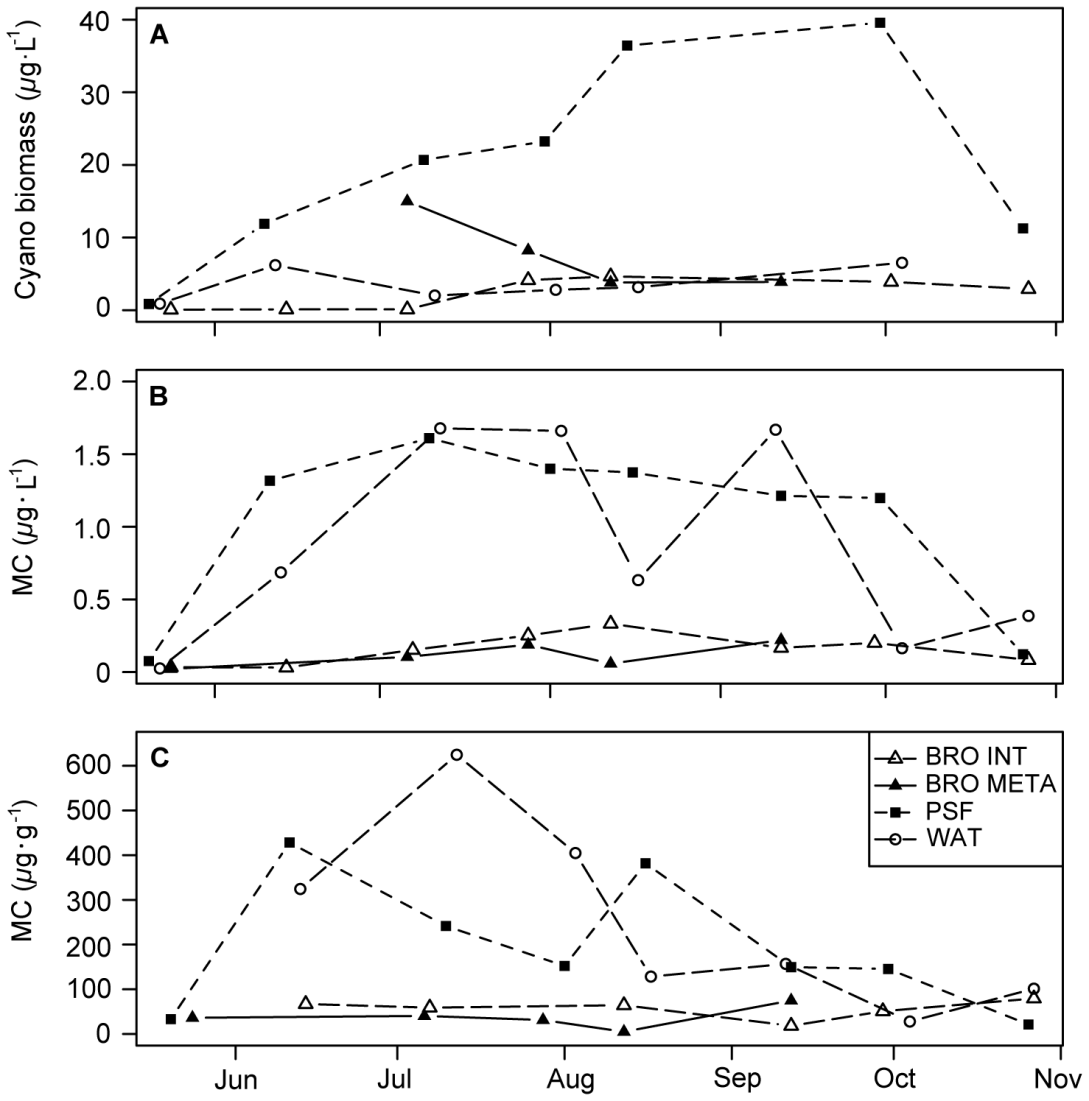
Cyanobacterial biomass and microcystin concentrations from May to October in the three study lakes. A) Cyanobacterial biomass (*μ*g·L^−1^); and total microcystin concentration expressed as B) *μ*g·L^−1^ and C) *μ*g·g^−1^ dry weight.

Mean total phytoplankton biomass was 3.4 and 8.4 mg·L^−1^ in the epilimnion and metalimnion of Bromont, respectively. Means were similar in Waterloo (4.1 mg·L^−1^), but five times higher in hypereutrophic PSF (23 mg·L^−1^). Cyanobacteria were the dominant phytoplankton (by biomass) on most sampling dates, generally comprising 90% of mean total biomass in all lakes with a minimum of 67% in the epilimnion of Bromont early in the season. PSF consistently had the highest overall cyanobacterial biomass (Fig. 2A).

The structure of the cyanobacterial communities varied among lakes (Table 2) and through time. Waterloo and PSF had similar taxonomic composition, although relative biomasses did differ between taxa. Several cyanobacteria species were found uniquely (e.g. *Anabaena smithii, Planktothrix agardhii,* and *Spirulina* spp.) or at much higher biomasses (e.g. *Anabaena planktonica*) in Bromont. Conversely, *Microcystis wesenbergii* was dominant in hyper-eutrophic PSF and found only at very low levels in Waterloo in one month (August). *Microcystis aeruginosa* was only identified in Waterloo where it contributed significantly to cyanobacterial biomass (Table 2). High biomass of *Planktothrix agardhii* was detected in Bromont and Waterloo. *Anabaena* spp. and *Aphanizomenon flos-aquae* were present in all lakes, but more commonly found in PSF and Bromont than in Waterloo.

**Table 2:** Average biomass (*μ*g·L^−1^) and mean percent of total cyanobacterial biomass (% cyano) of the dominant cyanobacterial taxa observed in the three study lakes across all sampling dates.

Mean species biomass	Bromont epilimnion		Bromont metalimnion		Waterloo		Petit Lac St. François	
(*μ*g·L^−1^)	Biomass	% cyano	Biomass	% cyano	Biomass	% cyano	Biomass	% cyano
Anabaena crassa*	1.4	0.01	ND	–	218.3	6.1	298.2	1.5
Anabaena planktonica*	206.9	9.1	253.3	3.3	69.9	1.9	ND	–
Anabaena smithii*	222.3	9.8	550.5	7.1	ND	–	ND	–
Anabaena spiroïdes*	ND	–	ND	–	660.3	18.3	ND	–
Aphanizomenon flos-aquae* ^§^	491.1	65.6	1,081.0	14.0	26.0	0.7	2,497.8	12.2
*Microcystis aeruginosa*	ND	–	ND	–	900.4	25.0	ND	–
*Microcystis* spp.	6.3	0.3	27.05	0.4	1,706.1	47.4	5,403.5	26.3
*Microcystis wesenbergii* ^§^	ND	–	ND	–	20.8	0.6	12,363.9	60.1
*Planktothrix agardhii*	342.6	15.1	5,700.2	73.7	ND	–	ND	–
*Spirulina* spp.^§^	2.3	0.1	120.12	1.6	ND	–	ND	–

ND, not detected; Rare species with low biomass that were observed <3 times are not listed and were not used in the statistical analyses. Species marked with * are potentially nitrogen-fixing, and those with ^§^ are considered non MC-producing.

### Total Microcystin Concentration

Total MCs were detected by ELISA on every sampling occasion at all locations (Fig. 2B). Total concentrations ranged from 0.06 *μ*g·L^−1^ in meso-eutrophic Lake Bromont to 1.68 *μ*g·L^−1^ in eutrophic Waterloo. In PSF, the maximum toxin concentration was measured in mid-July, while in Waterloo (the second most eutrophic lake) high values occurred in late July to early August and in mid-September. Waterloo and PSF displayed similar seasonal patterns and overall MC concentrations, despite the clear differences in both nutrient concentrations (Fig. 1A–F) and cyanobacterial biomass between the two lakes (Fig. 2A). This resulted in a much higher dry weight-specific MC content in Waterloo, reaching a maximum of 624.67 *μ*g·g^−1^ compared to 428.27 *μ*g·g^−1^ in PSF (Fig. 2C). In Bromont, dry weight-specific MC content was quite low throughout in the sampling period in both the epilimnion and the metalimnion (max. 79.40 and 74.92 *μ*g·g^−1^ respectively).

We used a multiple linear regression approach with forward selection based on AIC to determine if total MC concentration could be predicted from environmental variables and cyanobacterial species biomass. Highly correlated (*r*>0.60) explanatory variables were excluded from further analysis; TP and *k* were eliminated as both were most often highly correlated with other variables. Furthermore when TP was included in forward selection it was not retained as an explanatory variable. The final list of variables included in the analyses was: TN, TN: TP ratio, SRP, NO_3_^−^+NO_2_^−^, NH_4_^+^, DON, and water temperature (t°). The best model using environmental variables indicated a positive influence of DON, NH_4_^+^, and water temperature on total MC concentration (*R^2^_adj._* = 0.61; *p*<0.001, n = 24):
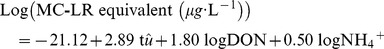
(Eq.1)


Variation partitioning demonstrated that DON, water temperature, and NH_4_^+^ explained 33%, 16%, and 11% of the variation in MC concentration respectively.

No significant multiple regression model emerged when we used only species-level cyanobacteria biomasses (as in Table 2) as explanatory variables although *Microcystis* spp. alone emerged as a strong predictor (*R* ^2^ *_adj._* = 0.63; *p*<0.00001, n = 24). When both the environmental and species variables were combined, the following model predicted 73% of the variation of the observed MC concentration (*R^2^_adj._* = 0.73; *p*<0.0001, n = 24):
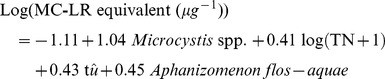
(Eq.2)


This final regression constituted the best model to predict total MC across the study lakes (based on R^2^_adj._). Variation partitioning demonstrated that *Microcystis* spp. explained the most variation (31%) followed by TN (12%). Although *A. flos-aquae* and water temperature both had a significant effect on the model they explained a relatively small portion of total variation (5 and 3% respectively).

### Microcystin Congener Composition

**Figure 3:**
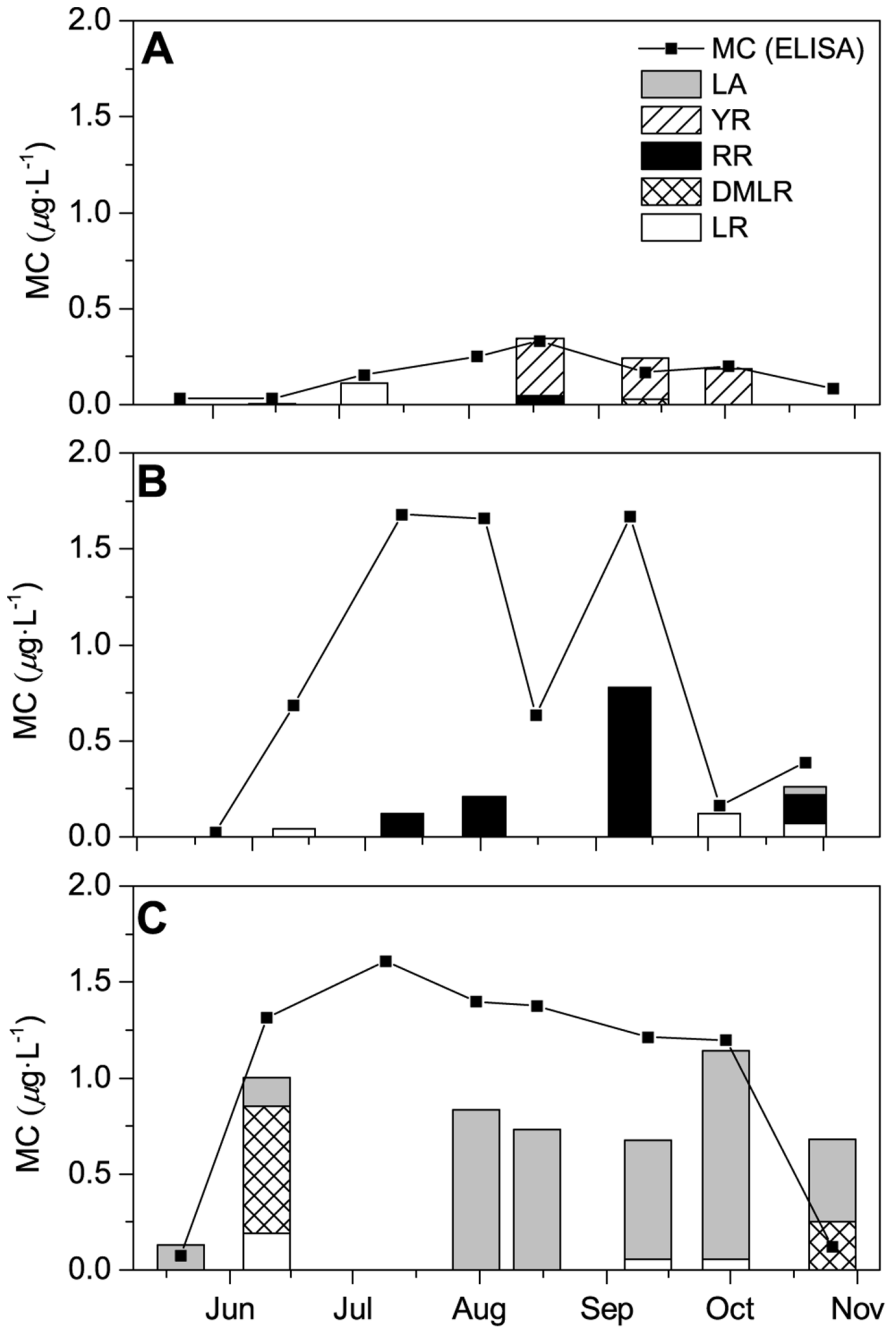
Temporal variation of total MC concentration measured by ELISA (full squares) and MC congeners measured by HPLC (stacked bars), from May to October in the three study lakes. A) Lake Bromont, B) Waterloo, and C) Petit Lac St. François.

The dominant MC congeners as detected by HPLC-UV varied both within and among lakes (Figs. 3A–C) over time. MC-LA was only found in PSF whereas MC-7dmLR and -YR were only detected in Bromont. MC-RR was present in Bromont and dominant in Waterloo, while MC-LR was present in all lakes. MCs in meso-eutrophic Bromont were mainly comprised of MC-YR although early in the season the dominant variant was -LR, switching to -YR in August and for the rest of the sampling season.

**Figure 4:**
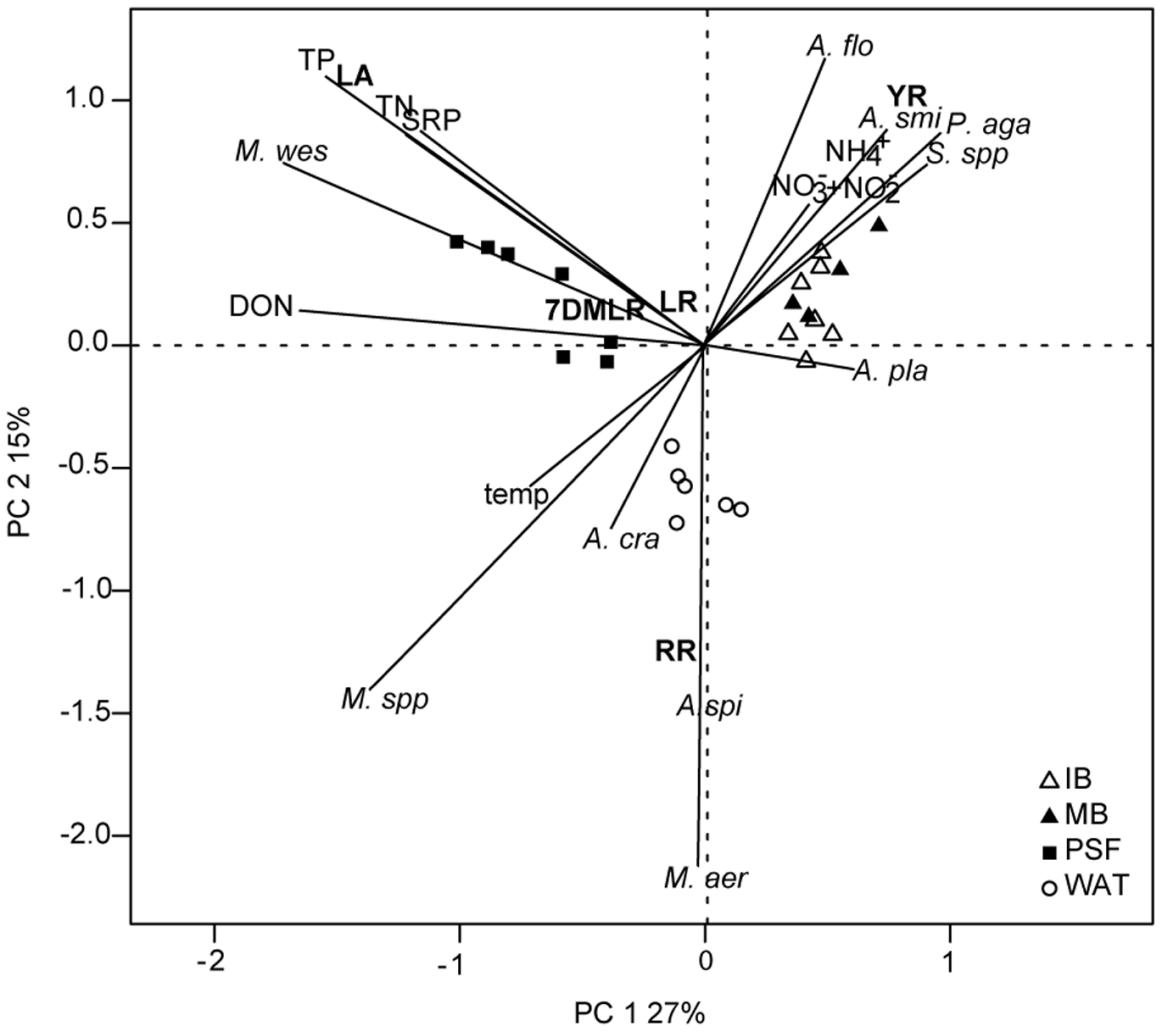
Principal component analysis (PCA) (scaling 1) of environmental variables, cyanobacteria species and MC congeners. The length of a vector is proportional to the importance of the descriptor to the sites. (IB; Bromont integrated epilimnion, MB; Bromont metalimnion, PSF; Petit Lac St. François, WAT; Waterloo). Cyanobacterial species are in *italics*, MC variants are in **bold**. *A. cra; Anabaena crassa, A. pla; Anabaena planktonica, A. smi; Anabaena smithii, A. spi; Anabaena spiroïdes, A. flo; Aphanizomenon flos-aquae, M. aer; Microcystis aeruginosa, M. wes; Microcystis wesenbergii, M.* spp.; *Microcystis* spp., *P. aga; Planktothrix agardhii, S.* spp.; *Spirulina* spp.

The PCA correlation biplot showed distinct patterns in the distribution of environmental descriptors, cyanobacteria species and MC composition among lakes, with sites well separated in the ordination space (Fig. 4). Variables that are close together are considered highly positively correlated, whereas those that are completely opposite to one another are negatively related. The first axis (PC 1) was mainly described by DON and explained 27% of total variation, whereas the second axis (PC 2) was mainly related to cyanobacterial community structure, with *Microcystis aeruginosa* and *Anabaena spiroïdes* dominance in Waterloo contributing another 15% of total variation. Lake PSF was associated with *Microcystis wesenbergii* and with indicators of nutrient enrichment (TN, TP, DON). Several other cyanobacterial taxa such as *Planktothrix agardhii*, *Aphanizomenon flos-aquae,* and *Anabaena smithii* were dominant in Bromont. The most important environmental descriptor in Bromont was water temperature, which was negatively related to other variables. Each lake was characterized by a dominant MC congener: MC-LA, -RR and -YR emerged in PSF, Waterloo and Bromont respectively. This pattern suggests an association of the MC congener composition with the community structure and environmental conditions of the different systems.

**Figure 5:**
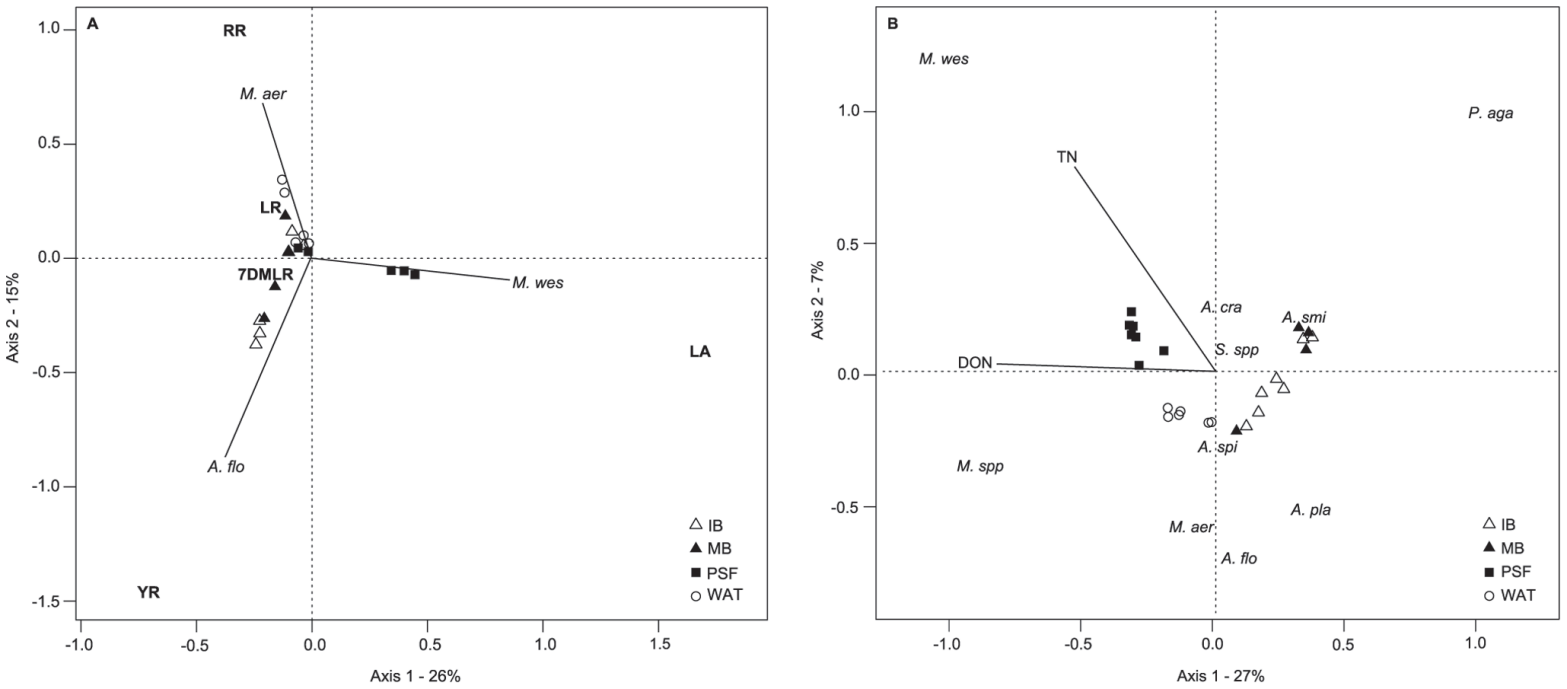
Redundancy analysis (RDA) of environmental variables, cyanobacteria species and MC congeners. **RDA** performed with forward selection by permutation (n-perm = 999) on A) Species biomass as explanatory variables for MC congener composition (*R^2^_adj._* = 0.40, *p* = 0.001) where combined *Microcystis aeruginosa, Microcystis wesenbergii and Aphanizomenon flos-aquae* explain 40% of the variation in MC congener concentration; and B) Environmental factors as explanatory variables for cyanobacterial species biomass (*R^2^_adj._* = 0.28, *p* = 0.001) where combined TN and DON explain 28% of the variation in species composition (for species abbreviations, see legend in Figure 4).

A series of redundancy analyses (RDA) with forward variable selection identified the factors potentially affecting MC congener composition in the lakes. We first determined the effect of environmental variables on congener composition, but the multivariate regression (RDA) was not significant (*p*>0.05; results not shown). Species composition however was significantly associated with MC congener composition (Fig. 5A). Together, *Microcystis wesenbergii, Microcystis aeruginosa* and *Aphanizomenon flos-aquae* explained 40% of the variation in congener composition across all lakes (*p* = 0.001, n = 24). According to the results of this RDA, *M. wesenbergii* was associated with MC-LA in PSF whereas *A. flos-aquae* was significantly related to MC-YR in Bromont and *M. aeruginosa* to MC-RR in Waterloo.

Given that environmental factors did not appear to affect MC congener composition directly, but that species did, we examined the relationship between environmental variables and cyanobacteria community structure using a third RDA (Fig. 5B). TN and DON were both positively related to the biomass of non-heterocystous cyanobacterial species and negatively related to heterocystous species (*R^2^_adj._* = 0.28). TN was closely associated with *M. wesenbergii* biomass (dominant species in PSF) and DON was related more to *Microcystis* spp. (dominant in Waterloo). TN was negatively related to *Anabaena planktonica* and *Aphanizomenon flos-aquae,* both potential N-fixers, in Bromont.

## Discussion

Several studies have shown how environmental factors affect cyanobacterial dominance and total microcystin concentration in lakes [22], [24], [25], but none have described the phytoplankton community dynamics in relation to the MC congener concentration and composition. In this study, multivariate analyses suggested that N concentrations and the various forms of N influenced the relative biomass of different cyanobacterial species and relationships with MC concentration and composition. There was no direct effect of environmental variables such as N availability on MC congener composition but only an indirect one mediated through cyanobacterial species composition.

### Cyanobacteria Biomass and Composition in Response to Environmental Factors

As is typical of eutrophic lakes [14], cyanobacteria dominated the phytoplankton communities in all three study sites. Cyanobacterial biomass was highest in the most eutrophic lake (PSF), where N and P concentrations were about eight times higher and where water temperature and pH, variables frequently associated with cyanobacterial dominance [47], [48], were also highest. The cyanobacterial species composition varied among systems (Table 2): the most common cyanobacterial taxa were non-nitrogen-fixing *Microcystis wesenbergii*, *Microcystis* spp., and *Planktothrix agardhii*, except in the epilimnion of lake Bromont where heterocystous N-fixing *Aphanizomenon flos-aquae* represented a significant fraction of the total biomass. N-fixing cyanobacteria were also present in all lakes. However, despite the low DIN during summer, heterocystous cyanobacteria represented only 15–25% of the total biomass in the two most eutrophic systems. Several studies have pointed to the lack of development of N-fixing taxa in lakes with apparent inorganic N limitation [49], suggesting other factors may constrain their development (e.g. low light, iron or other micronutrient limitation) and their ability to offset inorganic N deficits is questionable [15], [50].

The hypothesis that N availability and form influenced the cyanobacterial composition among lakes was largely supported. The multivariate models showed that relative biomass of *Microcystis* spp. and *Microcystis wesenbergii* was related to DON, whereas only the latter species appeared to respond to TN. Although it is known that phytoplankton rely mainly on DIN, DON constitutes the largest pool of fixed nitrogen in most aquatic systems [51] and could be an important source of N to these communities. Experimental studies have shown that DON enhances cyanobacterial development [28], [52] and more precisely, the growth of non-heterocystous taxa [53] several of which are toxin producers [21]. The DON pool is usually composed of refractory N-containing compounds as well as more labile molecules such as urea and dissolved free amino acids [54] both of which can be assimilated by cyanobacteria at relatively low cost [55].

Ammonium (NH_4_^+^) did not emerge as a significant variable in the multivariate model although it is considered important in structuring cyanobacterial communities based on empirical studies of some lakes [27], [30]. One possible explanation is that its concentration was both too low and relatively constant in these lakes, with the exception of the metalimnion in Bromont. Despite the low concentrations, NH_4_^+^ may have been an important source of N to the cyanobacteria through rapid recycling [56]. The PCA model (Fig. 4) did suggest that NH_4_^+^ was related to both heterocystous cyanobacteria (*Aphanizomenon flos-aquae*, *Anabaena* spp.), as well as non-N-fixing filamentous taxa (*Planktothrix agardhii* and *Spirulina* spp.) in lake Bromont. In contrast, the dominant taxa from lakes Waterloo and PSF were either not associated (*Microcystis wesenbergii*) or were negatively associated (*Microcystis aeruginosa* and *Microcystis* spp.) with NH_4_^+^, indicating that different taxa may respond in different ways to the concentration of this N-species in the environment.

### Microcystin Concentration Predicted from Environmental Variables and Species Composition

In this study DON concentrations explained a significantly large fraction of total MC concentrations (Eq. 1), with other specific N-forms (TN and NH_4_^+^) also emerging as important environmental explanatory variables in multiple regression models (Eq. 1 and 2). Interestingly, the highest total MC concentrations were observed in Waterloo where the DON accounted for more of the TN than in the other systems. We typically observed higher MC concentrations in relatively more NH_4_^+^-depleted and DON-replete waters (Waterloo and PSF), regardless of the total cyanobacteria biomass.

The biomasses of certain cyanobacteria species were also good predictors of MC concentration, with *Microcystis* spp. explaining almost a third of the variance again using multiple regression (Eq. 2). Others have similarly linked biomass composition and in particular *Microcystis* to MC concentration [22], [25] in North American lakes. In contrast, *Planktothrix agardhii* is the main producer of MC in lakes of northern Germany, rather than *Microcystis* [24]. All genera identified in this study, with the exception of *Spirulina* spp., *Aphanizomenon flos-aquae*, *Microcystis wesenbergii* and a few other rare species (data not shown), are known to be potential MC producers [18]. *Microcystis aeruginosa,* which is a known high MC-producer and one of the most widespread hepatotoxic species in freshwater [17], was only identified in Waterloo. Although *Microcystis aeruginosa* was not empirically related to MC concentrations, its dominance in Waterloo was associated with comparably high overall MCs with relatively lower cyanobacterial biomass resulting in the highest dry weight-specific MC content measured (635 *μ*g·g^−1^) in this study. This weight-specific MC concentration is similar to what has been observed in other eutrophic-hypereutrophic lakes with blooms primarily composed of *M. aeruginosa* [57]–[59]. *Planktothrix agardhii*, another well-known MC producer [24], was significantly correlated with MCs in lake Bromont but had a relatively low weight-specific MC content compared to literature values for this species [18].

The best overall model to predict MC concentration however combined both environmental variables and species biomass (Eq. 2). *Microcystis* spp. and TN emerged as the most important variables with *Aphanizomenon flos-aquae* and water temperature also explaining part of the variation again supporting the potential role of both N and species composition in influencing bloom toxicity. The positive influence of temperature is not surprising as several species of cyanobacteria are known to develop preferentially and have higher optimal growth rates in warmer waters [60].

### Microcystin Congener Composition

No environmental variables emerged as predictive variables to describe MC congener type and concentration, but cyanobacterial species composition and their relative biomass did. More surprisingly, with the exception of *M. aeruginosa,* the species showing significant associations with different MC congeners are not normally associated with MC-production: *Aphanizomenon flos-aquae* and *Microcystis wesenbergii.* The possibility that these species could also have toxic strains cannot be excluded from an evolutionary perspective [61]. However a study using both molecular and chemical approaches, demonstrated that *M. wesenbergii* was not a MC-producing species in Chinese waters [62] although it was often the dominant species in hepatotoxic blooms. This observation has been supported by other studies in European [63] and Japanese lakes [64] suggesting a community-level interaction such as facilitation. For example, high abundance of *Aphanizomenon flos-aquae* was observed prior to a toxic *Microcystis* bloom in Lake Mendota, where N-fixation by the non-toxic species was implicated in supporting the subsequent toxic cyanobacterial bloom phase [65]. Regardless, the main explanation for the differences in MC congener composition observed in this study was cyanobacterial species composition, whereby *Aphanizomenon flos-aquae* was related to MC-YR in Bromont, *Microcystis wesenbergii* with MC-LA in Lake PSF and MC-RR with *M. aeruginosa* in Lake Waterloo. MC congener composition is primarily strain-specific as opposed to species-specific but particular congeners appear more common in some regions than others suggesting some regional dominance of strains [18]. While the relative biomass of toxic and non-toxic cyanobacteria taxa influences the overall toxicity of the bloom [22], [25], our study further suggests that community structure affects the specific toxin congeners produced, which in turn would also influence overall toxicity.

Despite the lack of a direct association between congener composition and environmental variables, the forms of N present in lakes appeared to influence the cyanobacterial community composition and therefore indirectly, the dominant MC-congener type (Fig. 5B). There is growing evidence, at multiple scales of inquiry, supporting the role of N and N-speciation in influencing the presence and toxicity of cyanobacteria. In large-scale latitudinal studies, both temperature and N concentrations were the strongest explanatory variables for cyanobacterial biomass in lakes [66]. Our study is consistent with this pattern and supports both field and laboratory experiments that relate MC concentration to total nitrogen availability [24], [67], and water temperature [5], [68], [69]. However, the way in which nitrogen-speciation and availability shapes the cyanobacterial community structure may influence the type of MC congener that is produced thus influencing the overall toxicity. This study suggests a need to better understand N cycling within lakes and supports the recommendation to reduce N inputs along with P inputs to lakes [16], [24], [50], [70], in particular when the goal is to prevent the dominance of toxic cyanobacterial communities that pose a threat to human and wildlife health.
